# Excluded Lives: Migrant Status and Access to Healthcare in South Africa

**DOI:** 10.3390/ijerph23060775

**Published:** 2026-06-09

**Authors:** Alex Asakitikpi

**Affiliations:** Faculty of Humanities, The Independent Institute of Education, Johannesburg 1724, South Africa; aasakitikpi@emeris.ac.za

**Keywords:** healthcare access, South Africa, structural discrimination, medical xenophobia, black foreign nationals, undocumented migrants

## Abstract

**Highlights:**

**Public health relevance—How does this work relate to a public health issue?**

- The study demonstrates that discrimination as a social determinant of health requires conceptualizing it not merely as a discrete phenomenon but as a multi-level process embedded within broader systems of power and inequality, enabling a deeper understanding of how healthcare inequities are produced and sustained.
- The study highlights the importance of bureaucratic discretion as a mediating factor in accessing healthcare, contributing to the relatively limited attention paid to migration and health research in the Global South.

**Public health significance—Why is this work of significance to public health?**

- The study confirms the importance of extending healthcare access to marginalized populations such as migrants to reduce the spread of communicable diseases in society.
- The study demonstrates the urgent need for multi-level interventions within the context of public health to address discrimination as a structural determinant of healthcare inequities in South Africa.

**Public health implications—What are the key implications or messages for practitioners, policy makers and/or researchers in public health?**

- The findings of the study suggest that efforts to improve healthcare access in South Africa must address both policy frameworks and institutional cultures and practices that shape their implementation.
- The study supports the need for bridging the gap between constitutional guarantees and the lived realities of migrants by enforcing coordinated actions across policy, institutional, and community levels.

**Abstract:**

While South Africa’s Constitution guarantees the right to healthcare for all who live in the country, there are still inequities that affect vulnerable groups. Based on migration status, this paper examines how discrimination intersects with structural and institutional practices to produce unequal access to healthcare services for black foreign migrants and asylum seekers in South Africa. Desk reviews of policy frameworks, relevant academic literature, and documented case reports were used to analyze the disconnect that exists in South Africa’s rights-based legal commitments and the lived realities of foreigners in the country. Adopting a theoretical framework that integrates structural violence, intersectionality, and bureaucratic discretion, the findings are discussed by conceptualizing discrimination as a structural and interpersonal determinant of health. The findings suggest that the experiences of foreign nationals regarding access to healthcare services are not incidental but embedded within complex socio-political dynamics of scarce resources, institutional practices, and institutional ambiguity. The consequences of these inequities involve delayed care-seeking and increased vulnerability to preventable diseases among black immigrants, with a broader public health risk. Drawing from the study, policy clarity is recommended, and the strengthening of accountability mechanisms to ensure equitable access to healthcare in the country.

## 1. Introduction

South Africa’s Constitution is generally regarded as one of the most progressive in Africa, and for this reason, among other economic and political push factors across the continent, the country is a prime destination for migrants within the continent. However, the promise of a secure and non-discriminatory environment as enshrined in the Constitution soon evaporates as migrants begin to settle in the country. While some Black African migrants come into the country as skilled workers with legal documents, others enter the country as refugees and asylum seekers due to adverse conditions they face in their home countries. Although South Africa is signatory to international human rights frameworks, systemic inefficiencies significantly delay the processing of applications at the Department of Home Affairs for asylum seekers and refugees to improve their legal status in the country. The backlog of processing applications and regularization of migrants’ legal status creates conditions of precarity for migrants, which render them “illegal” in practice. This liminal status of migrants often shapes complex and at times strained interactions between migrants, citizens, and legal institutions. As a result of their migrant status and lived experience in the country, many Black African foreign nationals perceive themselves as unwelcome, both as legitimate and undocumented foreigners in the country [1]. This real or perceived negative perception is shaped by the way migrants are treated by host communities, as reflected in the ongoing anti-foreigner campaigns in South Africa (as of May 2026), during which black African immigrants have reportedly been subjected to harassment and physical violence [2,3,4]. In several instances, social interactions have often been characterized by derogatory remarks [5], verbal and physical abuse [6,7], and the restraint from accessing public institutions such as clinics and hospitals. The restriction or outright denial of migrants of basic social amenities does not only reflect human rights issues, but equally important, it reflects broader structural inequalities and entrenched socio-economic narratives that construct migrants as burdens or threat to social order in the country [8].

This study examines the ways in which migrant status, within the context of institutional and interpersonal discrimination, functions as a critical axis of exclusion within South Africa’s healthcare system. The study explores how migrants, with special focus on undocumented Black African foreign nationals, including refugees and asylum seekers, experience healthcare in the country, by examining how discrimination intersects with structural and institutional practices to produce unequal access to healthcare services for black migrants in the country. The study frames healthcare experience among migrants not merely as a service that is rendered or received, but as a contested terrain that is shaped by bureaucratic gatekeeping, institutional practices, and everyday forms of discrimination. These dynamics produce what can best be understood as “excluded lives,” where formal rights are overlooked or exploited by administrative barriers, facilitated by migrants’ fear of deportation, and xenophobic attitudes within healthcare institutions. The potential of the study is to inform policy and practice by highlighting various ways structural discrimination and institutional practices against foreign nationals may undermine equitable access to healthcare and their implications on marginalized groups and the South African public health system more broadly.

## 2. Literature Review

### 2.1. Discrimination as a Social Determinant of Health

The World Health Organization defines social determinants of health as “the conditions in which people are born, grow, live, work, and age, and the wider forces that shape the conditions of daily life” [9]. By broader structural forces the world body is referring to economic policies, social norms, political systems, legal frameworks, among other institutional structures that constitute the socio-political fabric of society. Discrimination, embedded within social institutions and displayed in everyday interactions, has been recognized as a key social determinant of health by scholars. For example, ref. [10] have not only acknowledged the relationship between discrimination as a social determinant of health but have stressed that discrimination must be understood not merely as an interpersonal phenomenon but as a structural process that systematically allocates resources, opportunities, and risks across populations. In the same vein, other scholars have emphasized that discrimination operates through multiple, overlapping levels, including interpersonal, institutional, and structural domains [11]. Interpersonal discrimination is pervasive in everyday dyadic interactions, involving individual prejudicial attitudes and behaviours that are displayed in formal and informal settings. At the meso-level, structural discrimination is embedded in institutions, and it manifests through organizational practices and norms that disadvantage certain groups, while at the macro-level, structural discrimination is the way in which laws, policies, and socio-economic systems create unequal access to resources and services [12]. These levels of discrimination are only theoretically dichotomous, in practice they are mutually reinforcing and collectively, they contribute to cumulative health disadvantages, especially when a subset of the population is targeted.

In the context of migration, discrimination has been conceptualized by scholars as a central mechanism through which inequities are produced. For example, ref. [13,14] has noted that many migrants often face intersecting vulnerabilities that are linked to legal status, language barriers, socio-economic marginalization, and limited access to social protection systems. Empirical studies have further shown that discrimination, including financial and administrative barriers, is a major impediment to healthcare access among migrants [15,16]. In such instances, these barriers limit access to preventive and curative services as well as contribute to cumulative poor overall health outcomes, including the spread of communicable diseases [17]. Importantly, the health impacts of discrimination in society extend beyond direct access to healthcare services. It has been reported to lead to chronic stress and reducing trust in public institutions among targeted populations from seeking services even in dire circumstances [18]. Within the context of public health, such circumstances have serious implications, especially in situations where early detection and treatment of infectious diseases, such as HIV and tuberculosis, are critical for population-level health outcomes.

### 2.2. Migration, Health, and Vulnerability

Another key determinant of health is migration, which has the potential of influencing both exposure to health risks and access to health services [13]. The “double burden” of the vulnerability of migrants’ experiences has been documented and examined within the context of healthcare access. Such vulnerability is more acute among undocumented migrants whose legal status is directly linked to their access to formal health systems. Globally, research has shown how migrants are less likely to access healthcare services compared to citizens, even when controlling for need [8]. Such disparity is often attributed to a combination of structural barriers, including lack of health insurance, restrictive migration policies, and fear of deportation [19,20,21]. In addition to these structural barriers, migrants may lack knowledge of available services or face local language and cultural barriers that hinder effective communication with healthcare providers [22,23]. In Sub-Saharan Africa specifically, migration dynamics are shaped by regional inequalities, economic disparities, and political instability. Because South Africa is one of the region’s most economically developed countries, it attracts a significant number of migrants from neighboring countries and across the continent. However, this influx has been accompanied by increasing public and political contestation over “uncontrolled migration,” often framed in terms of competition for scarce resources such as jobs, housing, and healthcare [24]. These tensions have contributed to the emergence of exclusionary attitudes and policies that negatively affect migrant populations in the country, especially within the context of healthcare services.

### 2.3. South African Policy and Legal Framework

South Africa’s legal and policy framework is often regarded as progressive because of its commitment to human rights and promise of social justice for all who live within its borders. The Constitution guarantees the right to healthcare services for “everyone,” while national legislation, including the National Health Act and the Refugees Act, affirms the entitlement of refugees and asylum seekers to access basic healthcare. In principle, these provisions position South Africa as a leader in the promotion of inclusive health systems in the Global South. However, existing literature highlights a significant gap between these legal commitments and their practical implementation. Recent research has consistently suggested that South Africa is experiencing a shift toward more exclusionary health policies, particularly in relation to undocumented migrants. For example, policy developments such as the National Health Insurance Act and immigration reforms have been criticized for institutionalizing exclusion and narrowing access to healthcare for non-citizens [25]. This shift is concerning as it reflects a broader trend toward the securitization of migration, in which access to social services is becoming increasingly tied to legal status and citizenship, which contradicts the constitutional and legal guarantees. The ambiguity and inconsistency of emerging policy frameworks and practice are increasingly exacerbating the challenges faced by migrants in accessing healthcare services. As healthcare providers operate within a context that is contradictory regarding migrants’ legal entitlements, it inevitably leads to inconsistent practices across facilities. The lack of clarity therefore provides spaces for discretionary decision-making among frontline staff in the health sector, which can result in discriminatory outcomes, particularly for undocumented migrants and asylum seekers.

### 2.4. Medical Xenophobia and Health

Xenophobia has been a critical factor in shaping the experiences of migrants in South Africa, including their access to healthcare services, a phenomenon that scholars have termed “medical xenophobia” or “health xenophobia.” The term is used to describe the “discrimination that medical professionals and institutions may exhibit towards non-citizens, which include prejudice by everyday citizens towards refugees and migrants in healthcare settings” [26]. Beyond its physical manifestation, medical or health xenophobia is also regarded as “negative attitudes and practices of healthcare workers towards foreign nationals based on their national origins” [27]. Thus, while xenophobia is often associated with violent outbursts, it also manifests in more subtle forms of social exclusion embedded within everyday practices and institutional settings. These forms of “everyday xenophobia” can be difficult to address, as they become normalized and routinized within organizational cultures. As observed by [28], the relationship between xenophobia and health is complex and multifaceted. On the one hand, xenophobic attitudes mostly lead to discriminatory practices that directly limit access to healthcare services by the target population. On the other hand, the broader climate of hostility and exclusion directly have effects on health by increasing stress, reducing social support, and discouraging engagement with public institutions. These dynamics are particularly pronounced among Black African migrants, whose experiences are shaped by the intersection of race, nationality, and socio-economic status. The existing literature suggests that xenophobia in South Africa is closely linked to broader socio-economic and political dynamics, including high levels of inequality, unemployment, and competition for resources [29,30,31]. In this context, migrants are often scapegoated for structural and socio-economic problems thereby reinforcing exclusionary attitudes and practices both in everyday interactions and formal institutions. Within healthcare settings, these dynamics can manifest in diverse ways, including the prioritization of citizens over non-citizens or outright denial of services, even in situations where legal frameworks mandate equal access. Such antagonisms have been directed mostly to Black African migrants, a practice some scholars have termed Afrophobia [32,33].

### 2.5. Synthesis and Research Gap

The above literature review highlights some key themes. Firstly, discrimination is a central mechanism through which health inequities tend to be produced and sustained, particularly for marginalized populations such as migrants. Secondly, although South Africa’s legal framework is formally inclusive, gaps exist between policy and practice. These gaps are significant as they result in widespread exclusion of migrants from healthcare services. Thirdly, these exclusions are mostly shaped by the combining structural factors of institutional practices and interpersonal dynamics, which are embedded within broader socio-political contexts, characterized by xenophobia and resource constraints.

The growing body of research focusing on migration and health in South Africa notwithstanding, important gaps remain. For example, much of the existing literature focuses on either policy analysis or individual experiences, with limited integration of these perspectives. Furthermore, in most studies there are no nuanced analyses of how different forms of discrimination, including legal, institutional, and interpersonal relations, interact to produce health inequities. This study seeks to address these gaps by providing an analysis of migrant status, framed within the context of discrimination, as a social determinant of healthcare access for Black African foreign nationals in South Africa.

## 3. Theoretical Framework

**Figure 1:**
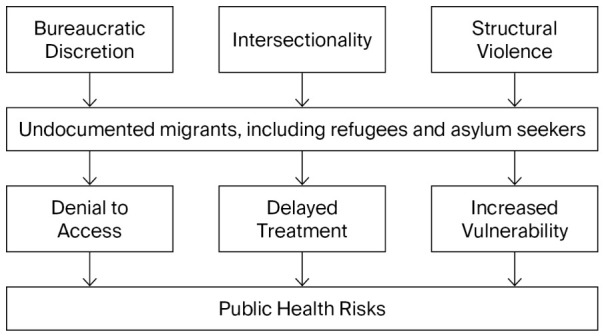
Conceptual framework of healthcare inequities of undocumented migrants in South Africa.

A multi-layered theoretical framework is adopted for this study, which examines how discrimination, based on migration status, shapes unequal access to healthcare services and ultimately how the dynamics affect public health in South Africa. The framework draws on three, but interrelated concepts of structural violence, intersectionality, and bureaucratic discretion to provide a nuanced understanding of how exclusion is produced and sustained within healthcare systems. These complementary perspectives provide a framework that enables an analysis that moves beyond individual experiences to interrogate the broader social, political, and institutional dynamics underpinning health inequities as shown in Figure 1 below.

### 3.1. Structural Violence and Health Inequities

The articulation of structural violence [34] and later expanded within public health by [35], provides a perceptive lens for understanding the systematic harm or disadvantage certain populations may experience within social structures. According to [36], structural violence focuses on “the social structures—economic, legal, political, religious, and cultural—that prevent individuals, groups and societies from reaching their full potential.” These institutional arrangements are conceptualized to be structured because they are embedded in social institutions and become “violent” because the outcome is harmful both individually and collectively. Within the context of healthcare systems, structural violence manifests through unequal access to services, discriminatory policies, and negative institutional practices against targeted vulnerable groups.

From a migration perspective, structural violence is activated in the ways legal frameworks, immigration policies, and health system constraints intersect to limit foreign nationals in accessing health services [11]. Undocumented migrants and asylum seekers face various barriers and practices, including demand of legal documents at point of service, fear of deportation, and limited institutional accountability, all of which function as mechanisms of structural violence, constraining their ability to access required healthcare services. Importantly, structural violence operates not only through overt exclusion but also through systemic neglect and under-resourcing of public health systems. Within the contexts of scarcity and competition for resources such as those in South Africa [28,30], healthcare providers may prioritize citizens over non-citizens, thereby reinforcing existing hierarchies, justifying the need to understand healthcare inequities not as isolated incidents but as outcomes of broader structural conditions.

### 3.2. Intersectionality and Migrant Status

Intersectionality offers a nuanced framework in analyzing how multiple forms of inequality interact at the individual and group levels. The seminal work of [37] on intersectionality emphasizes that social categories such as race, gender, and nationality are not discrete phenomena and therefore do not operate independently but intersect to produce unique experiences of advantages or disadvantages. In this study, intersectionality is appropriate in examining the experiences of Black African foreign nationals in South Africa. This target population occupies multiple marginalized positions as unwelcomed foreigners and is racially categorized within a broader historical context, shaped by apartheid and post-apartheid inequalities. As a result, their experiences of discrimination cannot be fully understood by examining migration status alone.

Intersectionality also provides insight into how institutional practices may affect subgroups differentially within migrant populations. For example, undocumented migrants or those awaiting their legal documents to be finalized by the Department of Home Affairs may face more severe barriers than recognized refugees. Furthermore, women migrants may encounter quadruple challenges related to gender-based discrimination in healthcare settings [14]. Foregrounding these intersecting identities allows for a comprehensive analysis of how discrimination operates in practice. In South Africa, where issues of race, nationality, and socio-economic inequality are interwoven, the categorization of individuals as “foreign” or “unwanted” carries implicit racial and cultural meanings, which reinforce the racialization of migration and sustain exclusionary practices within healthcare institutions [29].

### 3.3. Bureaucratic Discretion and Institutional Practices

Bureaucratic discretion is adopted in this study to examine how policies are implemented at the institutional level. Ref. [38]’s theory of “street level bureaucracy” proposes that frontline workers, including doctors, nurses, and supporting staff, play a critical role in shaping policy outcomes through their daily routine decisions and interactions with patients. In contexts where policies are ambiguous or resources are limited such as in South Africa, frontline actors may exercise considerable discretion in determining who receives services and under what conditions. Because healthcare workers operate within a complex environment characterized by competing demands, resource constraints, and unclear guidelines, the concept of bureaucratic discretion is significant [39]. For example, decisions about whether to provide care to undocumented migrants and asylum seekers may conflate personal attitudes, institutional norms, and perceptions of deservingness. Using their discretionary power, healthcare personnel can both mitigate and exacerbate inequalities. Sympathetic healthcare providers, on the one hand, may decide to offer service to undocumented patients despite administrative barriers, thereby promoting access. On the other hand, discriminatory attitudes or institutional pressures may lead to the denial of services by another healthcare worker, reinforcing exclusionary practices. In this way, bureaucratic discretion serves as a key mechanism through which structural inequalities are translated into everyday experiences among frontline staff.

### 3.4. Integrative Framework

Collectively, the above three theoretical perspectives provide a comprehensive framework to analyze migrant status as a social determinant of healthcare access. While structural violence focuses on the macro-level forces that shape access to resources and opportunities, intersectionality illuminates the multiple and overlapping forms of disadvantage experienced by migrants at the micro level. At the meso-level, bureaucratic discretion explains how these dynamics are enacted within institutional settings. By integrating these approaches as conceptualized in Figure 1, this study examines migrant status, framed within the concept of discrimination, not as a singular or isolated phenomenon but as a multi-level process that is embedded within broader systems of power and inequality. Figure 1 shows how bureaucratic discretion (involving frontline decision-making, ambiguous policies, and provider attitude to migrant patients), intersectionality (involving race, nationality, and legal status of migrants), and structural violence (systemic barriers, legal and policy constraints, and resource scarcity) interact to produce health inequities in South Africa. This framework enables a perceptive understanding of how healthcare inequities are produced and sustained and simultaneously provides a foundation for understanding the public health implications and identifying interventions that address both structural and institutional drivers of exclusion.

## 4. Research Methodology

### 4.1. Research Design

The study adopted a qualitative, desk-based research design to examine how discrimination of foreign nationals of African descent, based on their migration status, shapes their unequal access to healthcare services in South Africa. To achieve this objective, a desktop qualitative research approach was used for the study, based on the study focus to understand the complex social processes, institutional practices, and the lived experiences of migrants that cannot be fully captured through a quantitative survey [40]. Based on this rationale, the study used a critical interpretive approach to analyze how power relations, social structures, and institutional dynamics tend to influence how marginalized populations such as Black foreign nationals access healthcare in the country [41]. The research is exploratory and explanatory in nature as it sought to document patterns of healthcare exclusion and simultaneously providing a narration of the underlying mechanisms through which discrimination operates as a social determinant of health. Furthermore, the study integrated insights from policy analysis, empirical literature, and documented case studies that provided a comprehensive account of healthcare inequities that affect Black foreign migrants in South Africa.

### 4.2. Data Sources and Sampling Strategy

The selection of resources for the study was based on multiple secondary data sources. It drew on multi-source and interdisciplinary evidence-based data materials that were selected through a purposive sampling technique to ensure their relevance in achieving the research objectives [42]. The iterative and purposive search strategy involved the use of academic databases, such as Google Scholar and discipline-specific repositories, which were also complemented by targeted search of institutional websites, such as those of the World Health Organization, and Human Rights Watch. Thus, the key sources from which the study relied on include policy and legal documents such as the South African Constitution, the National Health Act, the Refugees Act, and the National Health Insurance policy, all of which speak to the national legislation and policy frameworks governing healthcare access and migration. In addition to these materials, the study also relied on government reports and official guidelines on healthcare provisions to provide a holistic picture of government position on healthcare access to all residents in the country. Additionally, peer-reviewed academic literature, including journal articles and scholarly article publications that focus on migration, health, discrimination, and social determinants of health, both globally and within the South African context were consulted. Search terms used in the selection of articles were combinations of keywords, including “migration,” “healthcare access,” “undocumented migrants,” “health policy,” “exclusion,” and “South Africa.” In the selection of materials, priority was given to recent studies (2015-2026) to capture contemporary dynamics, although legal and institutional documents outside this timeframe were also included due to their relevance to the study. Finally, to illustrate instances of healthcare denial, discrimination, or exclusion of migrants in South Africa, the study relied on media reports, including newspaper and television coverage [43]. These reported cases were used to contextualize and enrich the analysis. In selecting these eclectic materials for the study, inclusion criteria were guided by the study’s focus on Black African migrants with special focus on undocumented individuals such as refugees and asylum seekers, and their access to public healthcare services in the country. Sources that did not directly address the objectives of the study were excluded in the selection, review, and analysis. Data from selected sources were systematically charted by using a thematic extraction framework that captured type of sources, such as legal, academic, policy, and media, key arguments and findings, representations of migrants’ access to healthcare, identified barriers, and policy implications and recommendations. This methodological approach was deemed appropriate due to the fragmented and multidisciplinary nature of the study, which integrates legal, policy, and experiential knowledge in examining access to healthcare services among Black immigrants in South Africa.

### 4.3. Analytical Approach

The analysis of reviewed materials followed [44]’s thematic approach, which allowed for the systematic identification, analysis, and interpretation of patterns within the qualitative data generated. The analytical process followed four key stages. Firstly, an extensive reading of the selected materials was made to gain insight and a comprehensive understanding of contents. Following the familiarization with the data, initial coding of the key concepts identified was made, providing emerging patterns that relate to discrimination, healthcare access, and migration status. The emerging patterns led to the generation of codes that were both deductively and inductively derived (informed by the theoretical framework and emergence from the data respectively). The third stage of data analysis was the organization of the codes into broader themes that reflect the main dimension of healthcare inequities, such as documentation barriers, institutional discrimination, and xenophobic practices. Finally, the themes were analyzed in relation to the theoretical framework of the study by linking empirical findings to concepts such as structural violence, intersectionality, and bureaucratic discretion. In discussing these emerging themes, elements of critical discourse analysis were incorporated to explain migrants’ representation within policy documents and public narratives. By doing so, the study brought to bear the underlying tensions between authorities, citizens, and undocumented migrants of African descent, power relations, and discursive constructions that tend to shape access to healthcare based on migrant status in the country.

### 4.4. Ethical Consideration

Although the study is desk research that relied on secondary data, ethical considerations remained central to the research process, leading to the application for and approval of ethics waiver from the Independent Institute of Education Research Ethics Committee (Reference Number: CI00100420268). In addition to the ethics waiver, the study adhered to the key principles of respect of the study population, data integrity, responsible representation, especially with respect to the vulnerability of the study population. In this regard, the key ethical considerations included the confidentiality and anonymity of the research subjects, even though they are in the consulted materials and in the public domain. While using publicly available data, care was taken to avoid reproducing videos, or pictures that identify details of individuals in a way that could compromise their dignity or safety. Furthermore, the analysis avoided sensationalizing or stigmatizing migrant populations or citizens as well as mentioning the names of government officials directly linked to xenophobic utterances. Rather, it emphasized the structural and systemic factors that contribute to migrants’ marginalization. As much as possible, all consulted sources were critically evaluated to ensure reliability and validity, with preference given to peer-reviewed articles, reputable institutional publications, and mainstream news outlets. Finally, as a legal resident but a non-citizen in the country, the researcher acknowledged the potential influence of his positionality and maintained scientific analysis and transparency throughout the study.

## 5. Results

This section presents the key findings of the study. It organizes the findings around four interrelated themes that illustrate how discrimination, framed within the context of migrants’ legal status, operates as a social determinant of healthcare access for Black African foreign nationals in South Africa. The themes, as they emerged from the data analysis, included legal documentation as a gatekeeping mechanism towards healthcare access of the study population, institutional practices and everyday discrimination against undocumented Black foreign nationals in the country, xenophobia in relation to access to healthcare, and the consequences for health outcomes. Together, the findings highlight the multi-layered nature of exclusion within the healthcare system in the country. Finally, the analysis integrated the empirical evidence with the theoretical framework of structural violence, intersectionality, and bureaucratic discretion.

### 5.1. Constitutional and Legislative Framework to Healthcare

The findings of the study indicate that rights to healthcare in South Africa are enshrined in constitutional and legislative provisions. Section 27 of the Constitution of the Republic of South Africa (1996) guarantees that “everyone has the right to have access to health care services, including reproductive health care.” This provision is inclusive and applies to all individuals within the country’s borders, irrespective of nationality, legal status (documented or undocumented), race, or gender [45]. In Section 9(3), the Constitution further guarantees equal treatment of all who live in the country by stating that “The State may not unfairly discriminate directly or indirectly against anyone on one or more grounds, including… ethnic, or social origin.” In addition, the National Health Act 61 of 2003 provides legislative backing for the delivery of healthcare services, stipulating that all people, including foreign nationals, are entitled to access primary healthcare services at public health facilities such as clinics and community health centres [46,47]. These legal instruments collectively affirm South Africa’s commitment to equitable healthcare access for all people who live within the borders of the country.

### 5.2. Legal Documents and Access to Healthcare

Despite the progressive legal framework as presented above, the results of the study identified legal document requirements at health centers as a key barrier to healthcare access among black migrants in South Africa. The literature highlights how identification documents are frequently used as informal gatekeeping tools within healthcare facilities [1,48,49,50]. This practice is common with healthcare staff who often require patients to present identification documents, such as the national ID booklet or smart card, asylum permits, or proof of legal residence before receiving care. The inability to produce such documents from migrants means that they are often denied services or experience delays in receiving care. This stratified practice creates a contradiction between constitutional guarantees and lived experience of non-citizens due to their migration status. Thus, documentation operates as both an administrative requirement and a mechanism of exclusion that disproportionately affects vulnerable migrant populations. This key finding reveals that the implementation of constitutional and legal policies is inconsistent with the practice on the ground, providing room for subjective interpretation of the law and giving discretionary powers to frontline staff. For example, the literature has documented that in some health facilities, migrants have been able to access services without being asked to submit any form of documents, while in other facilities, strict enforcement of identification requirements leads to service denial and exclusion [51,52]. In this respect, migrants who are denied care and have limited financial resources to patronize expensive private healthcare services are less able to navigate the formal public administrative processes that define their identity and exacerbate their exclusion.

### 5.3. Institutional Practices and Everyday Discrimination

The findings further show that institutional practices and everyday interactions within healthcare settings contribute to the exclusion of migrants from healthcare services. Institutional practices include denial of treatment, excessive waiting times to see health personnel, informal fees charged by frontline staff, and differential treatment compared to citizens. Evidence from media reports indicates that migrants frequently encounter hostile attitudes. For example, documented accounts reveal instances where public officials have expressed exclusionary sentiments, including verbal abuse and instructing foreign nationals to go back to their home countries to receive treatment. In addition, civil society reports how vigilante groups obstruct access to healthcare facilities. Members of these groups have reportedly turned away patients of foreign nationalities from clinics in Jeppe and Hillbrow (Johannesburg) and Kalafong (Tshwane), often asserting that healthcare services should be reserved exclusively for South African citizens. These actions have led to physical intimidation and assault in some instances. As a result, migrants living with chronic conditions such as HIV, diabetes, and hypertension face significant interruptions in treatment, with serious implications for their health outcomes [53]. The literature also consistently documents patterns of discriminatory behaviour among some healthcare providers, including negative attitudes towards migrant patients and reluctance to treat undocumented individuals [15,27,48,54]. These findings suggest that exclusion is not only structural but also embedded in everyday clinical practices.

### 5.4. Medical Xenophobia

Xenophobia against the study population emerges as a central theme in the analysis. The intense hatred for foreigners, functions both as an institutional practice and as interpersonal interactions within healthcare settings. Although xenophobia is often displayed as an overt act of violence against migrants, the findings show its covert manifestations in everyday practices and attitudes among healthcare workers. These forms of ‘everyday xenophobia” are engrained within social and institutional contexts, thereby influencing how migrants are perceived and treated by citizens. Studies and reports [28,53,55,56,57,58] indicate that xenophobia manifests both at the systemic level and personal level. Medical xenophobia is reflected at the systemic level in policies and practices that indirectly exclude foreign nationals, and at the personal level xenophobia manifests in the negative attitudes and bahaviours of frontline workers within healthcare environments. One of the key cumulative effects of medical xenophobia is the erosion of equitable access to healthcare and the undermining of public health goals. Additionally, by discouraging health-seeking behaviour of migrants due to xenophobic sentiments, their wellbeing is not only compromised but poses broader risks to the larger population’s health, especially with regard to communicable diseases.

Together, the overall results of the study reveal a significant disconnect between South Africa’s progressive legal framework that guarantees health for all who live in the country and the lived realities of migrants’ access to healthcare. Although the South African Constitution and other national legislation guarantee universal access to healthcare without discrimination, multiple barriers undermine the realization of this goal.

## 6. Discussion

This study was designed to examine how migration status (framed within the context of discrimination) of undocumented Black African foreigners living in South Africa functions as a social determinant of unequal access to healthcare services in the country. The findings from literature reviews and other materials reveal that healthcare inequities are produced through a complex interplay of structural, institutional, and interpersonal factors, which reinforce existing scholarships that conceptualize discrimination as a multi-level determinant of health [10,11]. In this section, the findings are discussed within the conceptualized theoretical framework with the aim of highlighting their implications for our understanding of health inequities in both national and global contexts.

### 6.1. Discrimination as a Structural Determinant of Health

The findings from this study strongly support the argument that discrimination, rather than a discrete phenomenon, operates as a structural determinant of health, manifesting beyond individual and attitudinal level. Verbal abuse or differential treatments that characterize frontline staff and migrant patients’ interactions are significant not only at the interpersonal level but also at the institutional level. Discrimination is embedded within broader systems of exclusion, shaped by policy ambiguities, resource constraints, and socio-political dynamics. This interpretation aligns with the concept of structural violence, which emphasizes how institutional arrangements systematically disadvantage marginalized populations [35].

The persistence of legal document requirements as a gatekeeping mechanism in South Africa, is an illustration of how structural factors are translated into unequal healthcare. Although legal frameworks, including the Constitution, guarantee the right to healthcare for all, the implementation of these policies mediate administrative practices that effectively exclude undocumented migrants [52]. This disconnect between policy and practice reflects what has been described in the literature as “implementation gaps” in health systems, especially in resource-constrained settings, such as those in South Africa [59]. In addition, the findings suggest that structural discrimination is not always explicit but embedded in otherwise neutral policies and practices. As results of the study show, documentation requirements may be justified in terms of administrative efficiency or accountability, but they disproportionately affect migrants with precarious legal status, especially those whose applications for refugee status and asylum are pending at the Department of Home Affairs. This gap reflects the importance of critically examining how policies are operationalized and the unintended consequences they may produce.

### 6.2. The Role of Institutions and Bureaucratic Discretion

One of the key contributions of this study is highlighting the role of institutions and frontline staff in mediating access to healthcare. Healthcare facilities are found to be active sites of policy implementation where access is negotiated and contested. From a bureaucratic discretion perspective, it is evidential that frontline healthcare workers play a pivotal role in shaping outcomes for migrant patients [38]. The findings further show that the variability in policy interpretations and how they are applied across different facilities indicates the power of healthcare providers. While some providers act as gatekeepers by enforcing exclusionary practices based on documentation or perceived deservingness, others adopt inclusive practices. In other words, they act as barriers or facilitators that enable healthcare access. This dual role reflects the tension between institutional constraints and professional ethics, as healthcare workers tend to navigate competing demands in resource-constrained settings. Within this context, the role of bureaucratic discretion provides conditions of uncertainty and constraints, allowing health personnel to make decisions that can either facilitate or hinder access to care. These variations, which have been found to be embedded in institutional practices, highlight the importance of institutional culture and individuals in mediating access to care. These findings speak directly to the broader research on street-level bureaucracy that emphasizes policy outcomes as often determined not by formal rules but by the decisions of frontline actors. In the context of migration and health, the findings suggest that efforts to improve access must address both policy frameworks and institutional cultures and practices that shape their implementation.

### 6.3. Intersectionality and the Experience of Exclusion

Applying intersectionality lens in interpreting the findings provides insights into the differentiated experiences of migrants within healthcare systems. Among the study population, discrimination is not experienced uniformly across the country, rather experiences are shaped by the intersection of multiple social identities, including race, nationality, legal status, and socio-economic position. This complex interplay of factors supports existing literature that highlights the importance of intersectionality in understanding health inequities [37]. In the South African context, experiences of Black foreign nationals in the country are specifically shaped by the intersection of race and nationality within a broad socio-historical context, characterized by the apartheid regime and its enduring legacies. Although Black foreign nationals share a common racial identity with majority of South Africans, undocumented migrants of African descent are often constructed as “outsiders” or “foreigners,” and are specifically targeted in exclusionary practices that reflect xenophobia, or what some scholars have referred to as Afrophobia [32,33,60]. This understanding challenges the often-simplistic narratives of racial solidarity by highlighting the complexity of social hierarchies in post-apartheid South Africa, defined not only by racial features but also by nationality. Furthermore, the findings also highlight the vulnerability of undocumented migrants and asylum seekers, whose precarious legal status, defined by legal documentation, exacerbates their exclusion from healthcare services. These intersecting vulnerabilities create what can be described as “layers of disadvantage,” whereby multiple forms of discrimination interact to produce compounded health inequities [61,62]. To address these inequities, there is therefore a need to target interventions that recognize and respond to the specific needs of different subgroups within migrant populations.

### 6.4. Xenophobia, Belonging, and Health

In understanding the role of xenophobia in shaping access to healthcare, the study makes an important contribution in nuanced ways. While previous research has documented the prevalence of xenophobic attitudes in South Africa [29,63], this study further highlights how these attitudes translate into everyday practices within healthcare settings. Xenophobia operates not only through overt acts of violence but also through subtle forms of exclusion that are embedded within institutional norms and interactions. The construction of migrants as “undeserving” or as a “burden” on public resources plays a key part in legitimizing exclusionary practices among frontline staff and citizens. This social construction reflects the broader socio-political narratives that frame migration in terms of scarcity and competition, particularly in contexts of high inequality and unemployment [64,65]. Within the healthcare environment, these narratives contribute to the prioritization of citizens over non-citizens, even in situations where legal frameworks mandate equal access. Equally important, the findings suggest that xenophobia has both direct and indirect effects on the health of targeted populations, as it leads to discriminatory practices that limit their access to healthcare services, and simultaneously shapes their perceptions of the healthcare system, leading to fear and avoidance of care. This aligns with existing research on the health impacts of discrimination, which highlights the role of psychological factors in shaping health outcomes [18]. While healthcare discrimination is often examined within the context of South Africa and the Global South, it remains a worldwide issue. Similar patterns of inequitable treatment, bias, and exclusion have been documented in numerous high-income countries, highlighting the systemic and transnational nature of healthcare discrimination.

### 6.5. Implications for Public Health and Health Systems

The findings of the study have significant implications for public health and the functioning of health systems. The underlying factors associated with the exclusion of migrants from healthcare services do not only undermine government efforts to achieve universal health coverage but also exacerbate health inequities within the population. As the World Health Organization [6] has consistently emphasized, health systems must be inclusive and equitable to be effective to achieve health goals. From a public health perspective, excluding migrants from accessing healthcare services poses risks to both individuals and the broader population. Infectious diseases, once unfettered for whatever reasons, do not respect legal or national boundaries, with the broader implication of hindering disease prevention and control efforts. This understanding highlights the importance of adopting a population health approach that recognizes the interconnectedness of health outcomes. Finally, the findings also emphasize the need for health systems to address the social determinants of health, including medical xenophobia associated with migrant status and discrimination of foreigners more broadly. This requires a shift from a purely biomedical model of care that focuses mostly on pathogens, genetics, and the physiology of diseases, to a more holistic approach that considers the social, economic, and political factors that shape health. In this regard, addressing discrimination in all forms and the hierarchical system of deservingness is not only a matter of human rights but also a critical component of effective public health strategy.

### 6.6. Key Contribution to Knowledge

This study makes several contributions to existing literature. Firstly, by integrating structural, institutional, and interpersonal perspectives, the study advances our understanding of discrimination as a multi-level determinant of healthcare access. Secondly, it highlights the importance of bureaucratic discretion as a mediating factor in accessing healthcare. This is an area that has received relatively limited attention in migration and health research, particularly in the Global South. Thirdly, by providing a context-specific analysis of South Africa, the study contributes to broader debates on migration, health, and social justice in Africa and the Global South more broadly. Furthermore, by foregrounding the experiences of Black African migrants in South Africa, the study challenges common narratives that overlook intra-Africa migration and its implications for health. In doing so, the study calls for greater attention to the dynamics of migration within the African continent.

Within the context of policy and practice implications, the study highlights the urgent need for multi-level interventions in addressing discrimination as a structural determinant of healthcare inequities. Bridging the gap between constitutional guarantees and lived realities requires effective and coordinated action across policy, institutional, and community levels. Thus, one of the central implications of this study from a policy perspective is the need for clear, consistent, and enforceable policy frameworks regarding migrants’ access to healthcare. While existing legislation affirms the right to healthcare for all, ambiguities in policy interpretation and implementation both at the institutional and personal levels create subjective space for exclusionary practices. For this reason, policymakers should prioritize the development of explicit national guidelines that clearly define the entitlements of migrants, regardless of their status, especially, in relation to emergency services, maternal health, and communicable disease treatment. Such policy clarification should be accompanied by harmonization across sectors, including the National Department of Health, the Department of Home Affairs, and the Department of Social Development, to reduce contradictions and administrative barriers. In addition, as enshrined in the Constitution, policies should explicitly prohibit discrimination based on nationality or documentation status and outline mechanisms for enforcement and accountability. There is also a need to critically assess health reforms, including the national health insurance scheme, signed into law in 2024, to ensure that they do not inadvertently institutionalize exclusion. Embedding the principles of equity and non-discrimination within these reforms is essential for safeguarding the overall health of citizens and advancing universal health coverage in South Africa.

### 6.7. Limitation of Study

Some limitations are associated with this study. Firstly, the reliance on secondary sources without any primary field data such as interviews, focus group discussions, or field observations, may not fully reflect the lived experience of the study population in real time. Secondly, the secondary sources, especially documented cases and reports, may involve reporting bias, as instances of discrimination that are not documented or publicized. Finally, the study population focused only on Black African migrants in the country without considering other non-African migrants. For these reasons, the findings of the study limit the generalizability to other migrant populations in the country. These limitations notwithstanding, the study is still important and relevant as it provides a robust and theoretically informed analysis of discrimination as a social determinant of healthcare access in South Africa. The study also lays an important groundwork for future research to incorporate primary data and comparative perspective in the study of migrants lived experiences in relation to healthcare services.

## 7. Conclusions

This study set out to investigate the disjuncture between South Africa’s rights-based legal commitments and the lived experience of non-citizens, specifically, Black African foreigners living in the country. Based on the findings, the paper discusses and highlight the emerging key issues, including institutional and interpersonal discrimination that serves as a structural determinant of health, the role of institutions and bureaucratic discretion as mediating factors in accessing healthcare by migrants, how intersectionality and differential experiences of migrants expose their exclusion within the healthcare system, and the role xenophobia plays in influencing migrants’ perceptions of belonging and shaping their access and utilization of healthcare services in the country. The analyses and discussion also highlight the gaps in policy and practice implications, demonstrating the inequities that exist between citizens and migrants in accessing healthcare. In sum, addressing the discrepancy between the lofty promise of the Constitution and other legal frameworks regarding the rights of all who live in the country to healthcare services and the lived realities of migrants in the country, policy reform is recommended to address this tension. Finally, there is a need to strengthen institutional cultures and encourage social transformation that will work in tandem in creating an inclusive health system that upholds the right and dignity of all individuals who live in the Republic of South Africa as enshrined in its Constitution.

## Data Availability

No new data were created or analyzed in this study.
